# In Comparison to Pathological Q Waves, Selvester Score Is a Superior Diagnostic Indicator of Increased Long-Term Mortality Risk in ST Elevation Myocardial Infarction Patients Treated with Primary Coronary Intervention

**DOI:** 10.3390/diagnostics11050799

**Published:** 2021-04-28

**Authors:** Maria Holicka, Pavla Cuckova, Katerina Hnatkova, Lumir Koc, Tomas Ondrus, Petr Lokaj, Jiri Parenica, Tomas Novotny, Petr Kala, Marek Malik

**Affiliations:** 1 Department of Internal Medicine and Cardiology, University Hospital Brno, Jihlavska 20, 625 00 Brno, Czech Republic; holicka.maria@fnbrno.cz (M.H.); cuckova.pavla@fnbrno.cz (P.C.); koc.lumir@fnbrno.cz (L.K.); ondrus.tomas@fnbrno.cz (T.O.); lokaj.petr2@fnbrno.cz (P.L.); parenica.jiri@fnbrno.cz (J.P.); kala.petr@fnbrno.cz (P.K.); 2 Department of Internal Medicine and Cardiology, Faculty of Medicine, Masaryk University, Jihlavska 20, 625 00 Brno, Czech Republic; marek.malik@imperial.ac.uk; 3 National Heart and Lung Institute, Imperial College of London, 72 Du Cane Rd, Shepherd’s Bush, London W12 0NN, UK; k.hnatkova@imperial.ac.uk

**Keywords:** primary percutaneous coronary intervention, Q wave, Selvester score, ST elevation myocardial infarction

## Abstract

The development of pathological Q waves has long been correlated with worsened outcome in patients with ST elevation myocardial infarction (STEMI). In this study, we investigated long-term mortality of STEMI patients treated by primary percutaneous coronary intervention (PPCI) and compared predictive values of Q waves and of Selvester score for infarct volume estimation. Data of 283 consecutive STEMI patients (103 females) treated by PPCI were analysed. The presence of pathological Q wave was evaluated in pre-discharge electrocardiograms (ECGs) recorded ≥72 h after the chest pain onset (72 h Q). The Selvester score was evaluated in acute ECGs (acute Selvester score) and in the pre-discharge ECGs (72 h Selvester score). The results were related to total mortality and to clinical and laboratory variables. A 72 h Q presence and 72 h Selvester score ≥6 was observed in 184 (65.02%) and 143 (50.53%) patients, respectively. During a follow-up of 5.69 ± 0.66 years, 36 (12.7%) patients died. Multivariably, 72 h Selvester score ≥6 was a strong independent predictor of death, while a predictive value of the 72 h Q wave was absent. In high-risk subpopulations defined by clinical and laboratory variables, the differences in total mortality were highly significant (*p* < 0.01 for all subgroups) when stratified by 72 h Selvester score ≥6. On the contrary, the additional risk-prediction by 72 h Q presence was either absent or only borderline. In contemporarily treated STEMI patients, Selvester score is a strong independent predictor of long-term all-cause mortality. On the contrary, the prognostic value of Q-wave presence appears limited in contemporarily treated STEMI patients.

## 1. Introduction

ST elevation myocardial infarction (STEMI) cases are traditionally classified as Q-wave and non-Q-wave MI [1]. The development of pathological Q waves has long been correlated with worsened outcome [2]. During the last decades, prognosis of STEMI patients has markedly improved by treatment changes from thrombolysis to primary percutaneous coronary intervention (PPCI) [3,4]. Nevertheless, even in PPCI treated patients, the development of pathologic Q waves has been related to worsened prognosis [5]. Previous autoptic studies [6] and more recent magnetic resonance imaging (MRI) investigations have shown that the presence of pathological Q wave correlates, as expected, with more extensive myocardial injury [7].

While the presence of ST segment elevation and the classification of Q-wave and non-Q-wave MI are standardly used in contemporary clinical practice, other recognised electrocardiogram (ECG)-based indices are reported and clinically utilised less frequently. Among others, these include the Selvester score, which was introduced, in its preliminary form, already in 1972 [8]. This score appears to offer accurate estimation of the infarct size based on Q- and R-wave durations and on R/Q and R/S amplitude ratios. Already in the 1980s, subsequent modifications of the score were rigorously validated by comparisons with autopsy-estimated myocardial infarct sizes [9] and have also been shown to carry prognostic information [10]. In PPCI treated patients, the Selvester score has been reported to be an independent predictor of adverse short-term outcome [11]. However, long-term prognostic value of the Selvester score in PPCI treated patients has not been reported.

Although newer diagnostic and imaging modalities are presently available, ECG remains a cornerstone for cardiovascular disease management. ECG acquisition is simple to arrange, inexpensive, and widely accessible. Consequently, the aim of the present study was to compare the predictive value of the presence of pathological Q waves with that of the Selvester score when applied to long-term mortality risk in STEMI patients treated according to the contemporary standards, that is, by both PPCI and aggressive pharmacotherapy.

## 2. Methods

### 2.1. Patient Population

An ECG-based project enrolled 300 consecutive patients with acute STEMI admitted to our hospital between 2012 and 2015. All enrolled patients were referred to the coronary catheterization laboratory with the diagnosis of acute STEMI, fulfilling the criteria for PPCI according to the contemporary and present standards [12,13]. Patients unable (in cardiogenic shock and/or unconscious on admission) and those unable or unwilling to sign an informed consent were excluded. All enrolled patients provided signed written consent. The study was conducted according to the guidelines of the Declaration of Helsinki and approved by the Ethics Committee of University Hospital Brno.

From the total of 300 enrolled patients, data of 283 consecutive STEMI patients (103 females) were analysed. The remaining 27 patients were excluded because of missing data owing to early discharge (*n* = 26) and because of consent withdrawal (*n* = 1).

### 2.2. Clinical Measurements

Body mass index (BMI) was calculated as BMI = W/H^2^, where W is the body weight in kilograms and H is the body height in metres. Troponin T (TnT) levels were measured 24 h after the chest pain onset (Troponin T high sensitivity assay, Roche Diagnostic, Basel, Switzerland). Brain natriuretic peptide (BNP) levels were assessed in the morning of the second hospitalisation day (Architect BNP assay, Abbott Laboratories, Chicago, IL, USA). Left ventricular ejection fraction (LVEF) was measured by echocardiography before hospital discharge.

### 2.3. Follow-Up

Data on all-cause mortality were retrieved from the nation-wide health insurance registry of the Czech Republic, which included records of all enrolled patients. Hence, using all-cause mortality as the study outcome events ensured that no patients were lost during follow-up.

### 2.4. ECG Assessment

At the catheterization laboratory admission, all patients had a 12-lead ECG (the “acute” ECG). Using this ECG, the localization of left ventricular MI was categorized as anterior (ST segment elevation in leads V1–V4), inferior (leads II, III, aVF), lateral (leads I, aVL, and/or V5–V6), or septal (leads V1–V2) [14].

Further analyses using ECGs recorded more than 72 h after the chest pain onset, but before hospital discharge (“72 h” ECG).

The presence of pathological Q wave was assessed in the 72 h ECGs (72 h Q). The fourth universal definition of myocardial infarction (4th UDMI) was used: The pathologic Q wave is any Q wave in leads V2–V3 ≥0.02 s or QS complex in leads V2 and V3; Q wave ≥0.03 s and ≥0.1 mV deep or QS complex in leads I, II, aVL, and aVF; or V4–V6 in any two leads of a contiguous lead grouping (I, aVL; V1–V6; II, III, aVF) [15]. The Selvester score (Supplemental Table S1) [9] was evaluated both in acute ECGs (acute Selvester score) and in 72 h ECGs (72 h Selvester score). ECGs with left bundle branch block were excluded. All the ECGs were evaluated by two advanced cardiology fellows who operated independently of each other. In cases of discrepancy, a senior cardiologist provided the final evaluation.

### 2.5. Statistics and Data Presentation

Continuous data are presented as mean ± standard deviation (SD) and as median with interquartile ranges (IQR). Categorical data are presented as absolute and relative incidences. Differences between continuous variables were compared using a two-tail two-sample *t*-test assuming different variances between compared samples. Two separate backwards stepwise regression analysis models were used to predict death with continuous variables dichotomized at population medians (“median model”) and at the following cut-off values (“conventional model”): age ≥ 70 years, TnT ≥ 3 μg/L, BNP ≥ 500 ng/L, LVEF ≤ 40%, BMI ≥ 25 kg/m^2^, acute Selvester score ≥ 6, and 72 h Selvester score ≥ 6. Time-dependent all-cause mortality probabilities were displayed by Kaplan–Meier curves and compared by the log-rank and chi-square tests. The Kaplan–Meier curves were calculated (together with their confidence bands based on 1000 bootstrap repetitions) in subpopulations defined by presence or absence of 72 h Q wave, presence or absence of acute Selvester score ≥6, presence or absence of 72 h Selvester score ≥6, and other clinical variables (age, BMI, TnT, BNP, and LVEF) dichotomized at population medians. The subpopulations dichotomized by medians of age, BMI, BNP, and LVEF were then further dichotomized by the addition of presence or absence of 72 h Q and presence or absence of 72 h Selvester score ≥6, respectively. Statistical analyses were performed using IBM SPSS Statistics for Windows, Version 25, and in Statistica package, Version 6.1. *p*-level *<* 0.05 was considered statistically significant.

## 3. Results

Clinical characteristics of the patient population are shown in Table 1. The PPCI procedure of infarction artery was successful in all investigated patients. No patients developed cardiogenic shock during hospitalization. At discharge, all patients were on acetylosalicylic acid, 255 patients (90.11%) were on ACE inhibitors/angiotensine receptor blockers, and 273 (96.47%) were on betablockers. During the follow-up of 5.69 ± 0.66 years, 36 (12.7%) patients died.

Altogether, 184 patients (65.02%) presented with 72 h Q waves. Acute Selvester score and 72 h Selvester score values were ≥6 in 126 (44.52%) and 143 (50.53%) patients, respectively. Differences of clinical characteristics are shown in Table 2.

The population medians used in the Cox regression analysis were as follows: age 62.4 years, BMI 27.97 kg/m^2^, Troponin T 2.25 μg/L, BNP 283.25 ng/L, and LVEF 55%. The results of both Cox regression models are shown in Table 3. There were little differences between the models although the population medians differed from the pre-specified dichotomies.

In univariable analysis, the presence of 72 h Q showed only weak predictive value. On the contrary, 72 h Selvester score ≥6 was a strong predictor of all-cause mortality when tested in univariable (hazard ratio (HR) 4.601, confidence interval (CI) 2.013–10.513, *p* < 0.001) or multivariable analyses in both models (HR 3.226, CI 1.376–7.576, *p* = 0.001 for the “median model” and HR 4.184, CI 1.792–9.804, *p* = 0.007 for the “conventional model”). In both multivariable models, only age was a stronger predictor of death than 72 h Selvester score ≥6.

**Figure 1:**
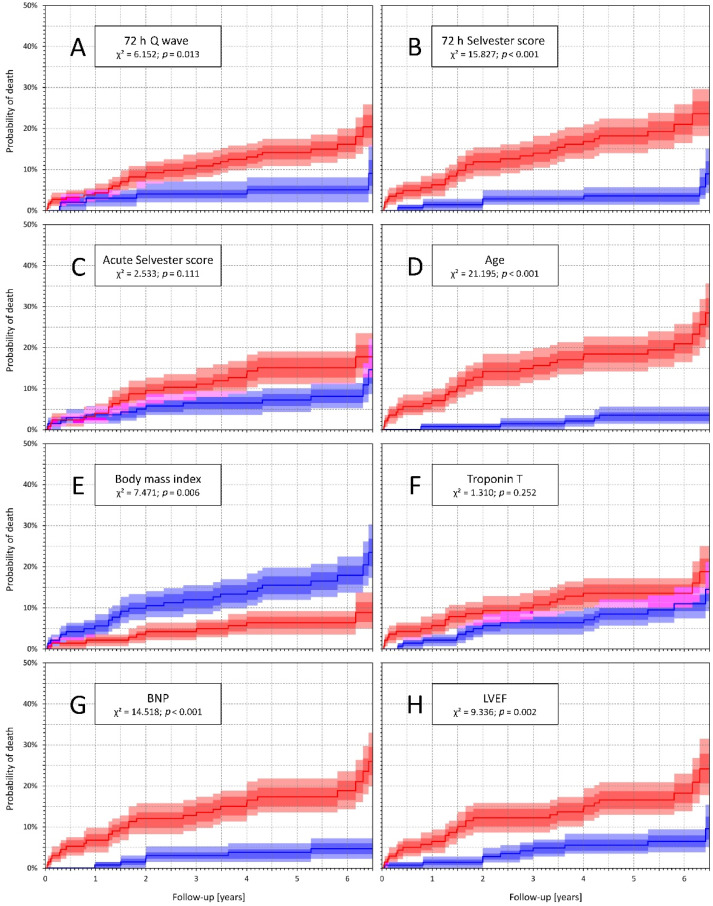
Kaplan–Meier analysis of probability of death in study patients. The panels (**A**–**C**) show comparisons of patients with (red line) and without (blue line) the presence of the following: panel (**A**)—pathologic 72 h Q wave; panel (**B**)—72 h Selvester score ≥6; panel (**C**)—acute Selvester score ≥6; panels (**D**–**H**) show differences according a value of a particular variable above (red line) and below (blue line) the median of the complete population: panel (**D**)—age; panel (**E**)—body mass index (BMI); panel (**F**)—troponin T (TnT); panel (**G**)—brain natriuretric peptide (BNP); panel (**H**)—ejection fraction of left ventricle (LVEF). In all panels, inter-quartile ranges are shown as the darker areas and 90% confidence intervals as the lighter areas.

**Figure 2:**
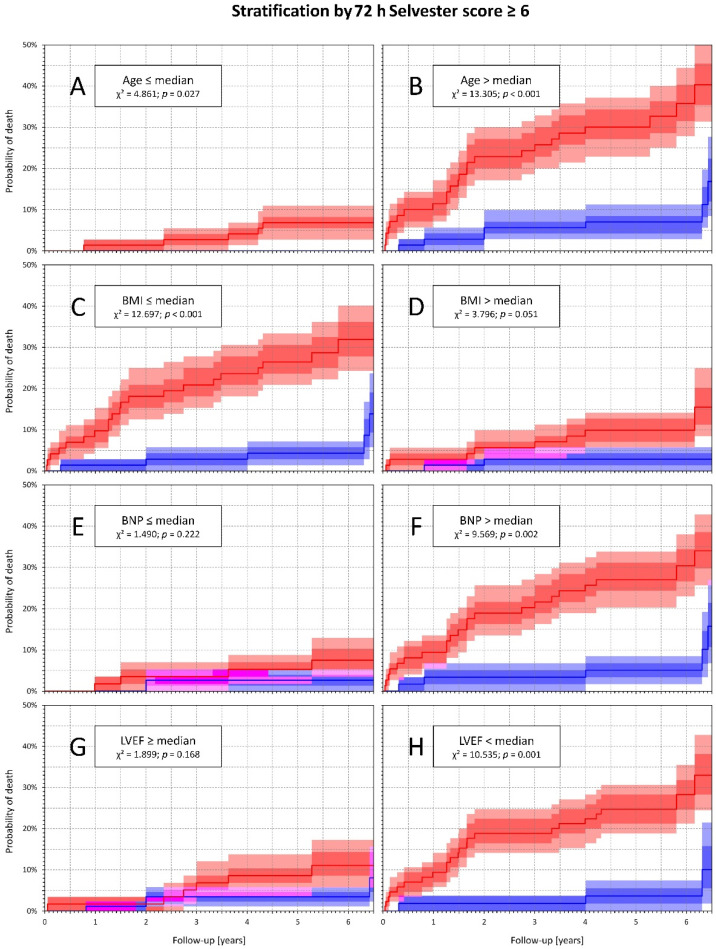
Kaplan–Meier analysis of probability of death in subpopulations defined by various clinical variables dichotomized at population medians and further dichotomized by the presence or absence of 72 h Selvester score ≥6. The panels show comparisons in particular subpopulations dichotomized by presence of 72 h Selvester score ≥6 (red line) or 72 h Selvester score <6 (blue line). Panel (**A**)—age below median, there were no deaths for age below median with 72 h Selvester score <6; panel (**B**)—age above median; panel (**C**)—BMI below median; panel (**D**)—BMI above median; panel (**E**)—BNP below median; panel (**F**)—BNP above median; panel (**G**)—LVEF above median; panel (**H**)—LVEF below median. In all panels, inter-quartile ranges are shown as the darker areas and 90% confidence intervals as the lighter areas.

**Figure 3:**
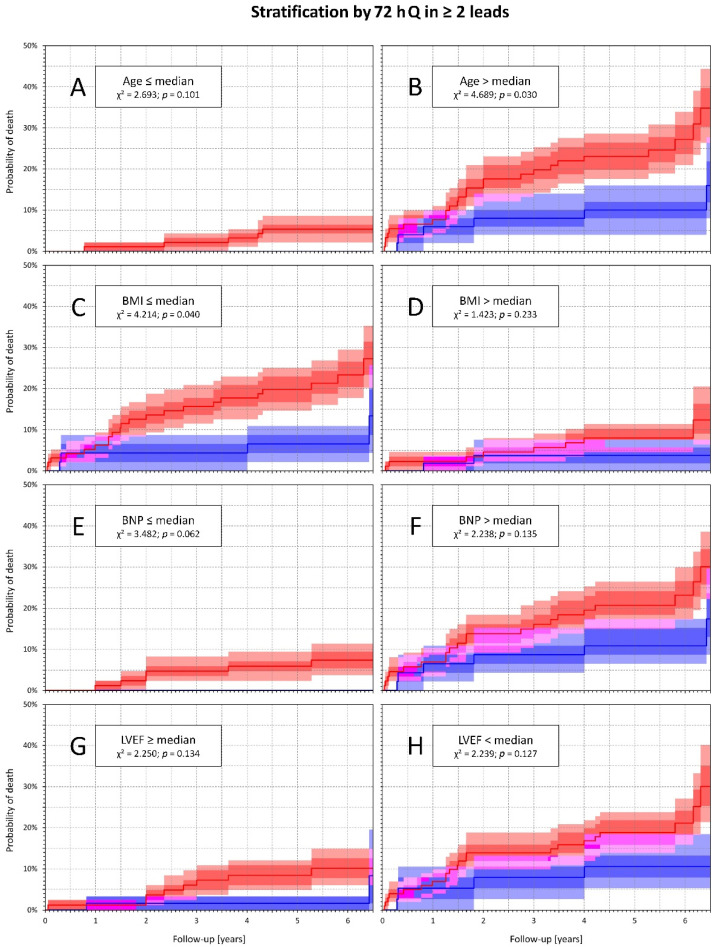
Kaplan–Meier analysis of probability of death in subpopulations defined by various clinical variables dichotomized at population medians and further dichotomized by the presence or absence of 72 h Q wave. The panels show comparisons in particular subpopulations dichotomized by the presence of 72 h Q wave (red line) or absence of 72 h Q wave (blue line): panel (**A**)—age below median, there were no deaths for age below median without 72 h Q wave; panel (**B**)—age above median; panel (**C**)—BMI below median; panel (**D**)—BMI above median; panel (**E**)—BNP below median, there were no deaths for BNP below median and without 72 h Q wave; panel (**F**)—BNP above median; panel (**G**)—LVEF above median; panel (**H**)—LVEF below median. In all panels, inter-quartile ranges are shown as the darker areas and 90% confidence intervals as the lighter areas.

The long-term total mortality was significantly higher in patients with 72 h Q compared with others (16.3% vs. 6.1%, *p* = 0.013). The difference in total mortality was even more pronounced when comparing patients with 72 h Selvester score ≥6 and <6 (20.3% vs. 5%, *p* < 0.001) (Figure 1). When 72 h Selvester score ≥6 was combined with BNP and age over median, BMI and LVEF under median the differences in total mortality were highly significant (*p* < 0.01 for all the variables) (Figure 2). On the contrary, if the same combinations were used with 72 h Q, the differences in total mortality were either absent (BNP over median, LVEF under median) or borderline (age over median, BMI under median) (Figure 3).

## 4. Discussion

In our population of contemporarily treated (PPCI + aggressive pharmacotherapy) STEMI patients, the 72 h Selvester score value ≥6 points was not only a strong predictor of long-term all-cause mortality risk, but it also had additive risk-stratification value when combined with other recognised outcome factors. On the contrary, the prognostic value of the presence of pathological Q waves was only limited.

The presence of pathological Q waves was firstly described by Harold Pardee in 1930 [16]. Since then, the definition of Q wave infarction has undergone several changes. Since the first universal MI definition (UDMI) of 2007 [17], the pathologic Q wave definition has been based mostly on its duration of at least 20 or 30 ms (Table 1). Importantly, 20 ms represents only 0.5 mm of ECG recordings with the standard recording speed of 25 mm/s, which places the definition at the border of human ability of interpreting and diagnosing customary paper printed ECGs. Traditionally, MI patients have been and still are conventionally classified as Q and non-Q MI, although the clinical utility of this distinction has repeatedly been challenged [18]. The reasons for these challenges include inconsistent methodology of Q wave definition (different versions of the so-called universal MI definitions were reported to result in Q-wave IM incidences ranging between 28% and 58% [7]) and doubtful pathologic basis of Q wave formation. The standard concept of a “window” of infarcted tissue was challenged by MRI and autopsy studies, which have shown that infarction scars are rarely homogenous and contain regions of viable tissue [19]. The changes in the definition of Q-wave MI have also made older and more contemporary studies mutually incomparable because they resulted in different incidences of Q versus non-Q MIs.

Despite these problems, a recent study reported association of Q-wave MI with larger infarct sizes assessed by cardiac MRI [7]. Another relatively recent study found that, in PPCI treated patients, the non-Q MI was an independent predictor of absent 1-year mortality [5]. Nevertheless, the authors of this study did not specify the Q-wave definition used.

We thus believe that our study contributes to the discussion of the prognostic value of Q-wave MI according to the 4th UDMI [15] in contemporary treated STEMI patients. Our data indicate that, using this definition, the presence of pre-discharge Q-wave MI has lost its predictive strength and became only a weak predictor of long-term mortality risk. Additionally, the presence or absence of pre-discharge (72 h) Q-wave MI pattern was not related to any substantial differences in total mortality in high-risk sub-populations defined by other clinical variables (see Figure 3).

The infarction myocardial damage appears to correlate more strongly with survival than with myocardial function assessed by LVEF [19,20]. This brings the importance of Selvester ECG score, which was developed and subsequently validated to estimate the extent of infarction. It has been shown to outperform other infarct scoring methods (e.g., Minnesota Score, Novacode, and Cardiac Infarction Injury Score) even for multiple infarctions [21,22].

Only limited data on the usefulness of Selvester score are available for PPCI treated patients. In a 90-day follow-up study, the score was a strong predictor of adverse outcome [11]. Our study extends the follow-up of PPCI treated patients and shows that the pre-discharge (72 h) Selvester score above or equal to 6 points (which was a median value in our data) is also a strong predictor of long-term total mortality in contemporarily treated STEMI patients. Furthermore, we were also able to use Selvester score to successfully and significantly sub-stratify high-risk subpopulations defined by other clinical variables (as shown in Figure 2). Interestingly, this also included patient stratification by the so-called obesity paradox observed in our study [23]. Combination of BMI ≤median and pre-discharge (72 h) Selvester score ≥6 identified high risk patients.

Note also that, in our study, total mortality was not different in subgroups dichotomized by TnT median value, and TnT value above the median was not an independent predictor of death. Possible explanations include generally low levels of TnT in the described group (median 2.25 µg/L), well below the 3.5 µg/L threshold that has recently been shown to offer an important predictor for substantial myocardial damage [24]. Moreover, exclusion of patients in cardiogenic shock at admission (according to the design of the source study) might have contributed.

Risk stratification is an important part of clinical practice and of clinical decision making. In that respect, Selvester score appears valuable for the identifications of patients with the highest risk who might be appropriate candidates for individually tailored aggressive pharmacotherapy (e.g., including repeated efforts to up-titrate ACE inhibitors and beta-blockers in hypotensive patients) [25]. As the assessment of Selvester score requires not more than a standard 12-lead ECG recording, the valuable risk-related information that it provides is widely accessible at minimal cost.

Limitations of our study also need to be considered. We describe a single centre experience. Patients in cardiogenic shock or unconscious at presentation were excluded according to the design of the source study. The number of investigated patients was relatively small, preventing many sub-group analyses. The follow-up considered only total mortality. Neither the classification of mortality modes nor data on other follow-up events such as stroke [26,27], re-infarctions [28], or heart-failure worsening [29] were available.

## 5. Conclusions

In contemporarily treated STEMI patients, the prognostic value of Q-wave appears rather limited. On the contrary, the Selvester score, which is a widely accessible and inexpensive ECG classification method, is a strong predictor of long-term all-cause mortality independent of other clinical risk factors.

## Figures and Tables

**Table 1:** Population characteristics (*n* = 283).

Sex (*n* = 283)	Women/Men	103 (36.4%)/180 (63.6%)
Age (years)		63.07 ± 11.91
		62.4 (IQR 54.90; 71.88)
BMI (kg/m^3^)		28.38 ± 4.59
		27.97 (IQR 24.87; 30.81)
Previous MI Previous PCI/CABG		23 (8.13%)
		27 (9.54%)
Hypertension Dyslipidemia Diabetes mellitus		170 (60.07%)
		160 (56.53%)
		61 (21.56%)
AMI localization *	Anterior	124 (43.81%)
	Inferior	138 (48.76%)
	Lateral	46 (16.25%)
	Septal	5 (1.76%)
	Posterior	10 (3.53%)
Q wave after 72 h		184 (65.02%)
TnT max. (ng/mL)	(*n* = 281)	3.36 ± 3.36
		2.25 (IQR 0.98; 4.98)
BNP (pg/mL)	(*n* = 266)	414 ± 406.66
		283.25 (IQR 153.23; 546.15)
LVEF (%) at discharge	(*n* = 282)	52 ± 10.19
		55 (45; 60)
Lenght of follow-up (years)		5.69 ± 0.66
		5.60 years (IQR 5.18; 6.22)
Total mortality		36 (12.7%)

* Multiple AMI localizations are possible. Where *n* < 283, lower counts due to missing values are presented. AMI = acute myocardial infarction, BMI = body mass index, CABG = coronary artery bypass surgery, PCI = percutaneous coronary intervention, BNP = brain natriuretic peptide, LVEF = left ventricular ejection fraction, IQR = interquartile range.

**Table 2:** Comparison of clinical characteristics of patients with and without Q wave and with 72 h Selvester score values of ≥6 and ˂6.

	72 h Q Wave Present (*n* = 184)	72 h Q not Present (*n* = 99)	*p* Value	72 h Selvester Score ≥6 (*n* = 143)	72 h Selvester Score ˂6 (*n* = 140)	*p* Value
Age	63.1 ± 12.2	63.0 ± 11.5	NS	63.1 ± 12.8	63.0 ± 11.0	NS
BMI	28.3 ± 4.6	28.6 ± 4.6	NS	28.5 ± 4.8	28.3 ± 4.4	NS
TnT (μg/L)	4.0 ± 3.7	2.2 ± 2.3	<0.001	4.2 ± 3.7	2.5 ± 2.8	<0.001
BNP (pg/L)	412.8 ± 365.2	416.3 ± 477.2	NS	454.5 ± 410.6	374.7 ± 401.9	NS
LVEF	50.6 ± 10.7	55.1 ± 10.0	0.001	49.7 ± 11.4	54.7 ± 9.2	<0.001

BMI = body mass index; BNP = brain natriuretic peptide; LVEF = ejection fraction of left ventricle; NS = not significant; TnT = troponin T.

**Table 3:** Predictors of death in STEMI patients treated with primary coronary intervention.

Variable	Univariable Analysis			Multivariable Analysis		
	*p*-Value	HR	95% CI of HR	*p*-Value	HR	95% CI of HR
Age above median	<0.001	6.780	2.636–17.443	<0.001	9.804	2.967–32.258
TnT above median	0.256	1.469	0.757–2.849			
BNP above median	0.001	4.756	1.962–11.528			
LVEF below median	0.004	2.956	1.425–6.132			
BMI above median	0.009	0.376	0.181–0.780	0.048	0.454	0.208–0.992
72 h Q wave present	0.018	2.885	1.200–6.936			
72 h Selvester Score ≥6	<0.001	4.601	2.013–10.513	0.001	4.184	1.792–9.804
Acute Selvester Score ≥6	0.116	1.750	0.870–3.519			
Age ≥ 70 years	<0.001	5.813	2.859–11.818	<0.001	4.950	2.237–10.989
TnT ≥ 3 µg/L	0.098	0.575	0.299–1.107			
BNP ≥ 500 ng/L	0.034	2.101	1.058–4.167			
LVEF ≤ 40%	<0.001	3.800	1.955–7.388	0.044	2.179	1.021–4.63
BMI ≥ 25 kg/m^2^	0.331	0.709	0.354–1.419			
72 h Q wave present	0.018	2.885	1.200–6.936			
72 h Selvester score ≥6	<0.001	4.601	2.013–10.513	0.007	3.226	1.376–7.576
Acute Selvester score ≥6	0.116	1.750	0.870–3.519			

BMI = body mass index; BNP = brain natriuretric peptide; CI = confidence interval; HR = hazard ratio; LVEF = ejection fraction of left ventricle; STEMI = myocardial infarction with ST elevations; TnT = troponin T.

## Data Availability

The raw data supporting the conclusions of this article will be made available by the authors.

## Supplementary Materials

The following are available online at https://www.mdpi.com/article/10.3390/diagnostics11050799/s1, Table S1. QRS scoring system for estimating infarct size—The Selvester Score.
