# Supplementary material for: In Comparison to Pathological Q Waves, Selvester Score Is a Superior Diagnostic Indicator of Increased Long-Term Mortality Risk in ST Elevation Myocardial Infarction Patients Treated with Primary Coronary Intervention
Source: Diagnostics (Basel). 2021 Apr 28;11(5):799. doi: 10.3390/diagnostics11050799 (PMC8146038; doi:10.3390/diagnostics11050799)
Supplement: Supplementary file 1 [file diagnostics-11-00799-s001.zip › diagnostics-1172386-supplementary.pdf]

Supplementary Materials

In Comparison to Pathological Q Waves, Selvester Score is a Superior Diagnostic Indicator of Increased Long-Term Mortality Risk in ST Elevation Myocardial Infarction Patients Treated with Primary Coronary Intervention

Maria Holicka [1,2], Pavla Cuckova [1,2], Katerina Hnatkova [3], Lumir Koc [1,2], Tomas Ondrus [1,2], Petr Lokaj [1,2], Jiri Parenica [1,2], Tomas Novotny [1,2,*], Petr Kala [1,2], and Marek Malik [2,3]

1 Department of Internal Medicine and Cardiology, University Hospital Brno, Jihlavska 20, 625 00 Brno, Czech Republic; holicka.maria@fnbrno.cz (M.H.); cuckova.pavla@fnbrno.cz (P.C.); koc.lumir@fnbrno.cz (L.K.); ondrus.tomas@fnbrno.cz (T.O.); lokaj.petr2@fnbrno.cz (P.L.); parenica.jiri@fnbrno.cz (J.P.); kala.petr@fnbrno.cz (P.K.)

2 Department of Internal Medicine and Cardiology, Faculty of Medicine, Masaryk University, Jihlavska 20, 625 00 Brno, Czech Republic; marek.malik@imperial.ac.uk

3 National Heart and Lung Institute, Imperial College of London, 72 Du Cane Rd, Shepherd's Bush, London W12 0NN, UK; k.hnatkova@imperial.ac.uk

* Correspondence: novotny.tomas3@fnbrno.cz; Tel.: +420-53223-2454

Supplemental Table 1. QRS scoring system for estimating infarct size - the Selvester Score (see Ref. 9)

Lead	Q duration (msec)	R duration (msec)	Ratios	Points	Lead max.
**I**	≥30			1	2
			R/Q≤1	1	
**II**	≥40			2	2
	≥30			1	
**aVL**	≥30			1	2
			R/Q≤1	1	
**aVF**	≥50			3	5
	≥40			2	
	≥30			1	
			R/Q≤1	2	
			R/Q≤2	1	
**V1**	any			1	4
		≥50		2	
		≥40		1	
			R/S≥1	1	
**V2**	any	≤20		1	4
		≥60		2	
		≥50		1	
			R/S≥1,5	1	
**V3**	any	≤30		1	1
**V4**	≥20			1	3
			R/Q or R/S ≤0,5	2	
			R/Q or R/S ≤1,5	1	
**V5**	≥30		R/Q or R/S ≤1	1	3
			R/Q or R/S ≤3	2	
				1	
**V6**	≥30			1	3
			R/Q or R/S ≤1	2	
			R/Q or R/S ≤3	1	

Lead max – the maximum point count for particular lead
